# Diabetes in axial spondyloarthritis: a systematic review and meta-analysis of observational studies

**DOI:** 10.1007/s00296-024-05700-7

**Published:** 2024-09-12

**Authors:** Leher Gumber, Harini Samarasinghe, Praveen Gladston, Arumugam Moorthy

**Affiliations:** 1 Translational and Clinical Research Institute, Newcastle University, Newcastle-upon- Tyne, UK; 2 Northumbria Healthcare NHS Foundation Trust, Northumberland, UK; 3 University Hospitals of Northampton NHS trust, Northampton, UK; 4 Wirral University Teaching Hospital NHS Foundation Trust, Wirral, UK; 5 Department of Rheumatology, University Hospitals of Leicester NHS Trust, Leicester, UK; 6 College of Life Sciences, University of Leicester, Leicester, UK

**Keywords:** Diabetes, Axial spondyloarthritis, Epidemiology, Medical research

## Abstract

Axial spondyloarthritis (axSpA) is a chronic inflammatory condition with an increased risk of cardiovascular disease (CVD). Diabetes is a well-established risk factor for CVD and stroke. The objective of this study was to conduct a systematic review to: (i) identify the prevalence of diabetes in axSpA and (ii) compare the risk of diabetes in patients with axSpA and without. A comprehensive literature search was performed for articles published between 1 January 2000 and 15 November 2023 using Medline, Embase and Scopus (PROSPERO: CRD42023482573). Observational studies reporting prevalence, incidence or risk of diabetes in axSpA were included. Search results were independently screened by at least two reviewers. Quality of included studies were assessed using the JBI critical appraisal tool. Study-specific proportions and odds ratios (OR) were combined in a random-effects meta-analysis. 2257 articles were identified from database searching from which 23 studies were included for analysis amounting to a combined sample size of 65 025 patients. The pooled prevalence of diabetes in people with axSpA was 7.0% (95% CI 5.9–8.0%; predictive interval 2.4–12.9%; *p* < 0.001). The funnel plot was symmetric suggesting no small-study effects (I^2^ = 98.1% (95% CI 0.05–0.08), τ^2^ = 0.02; *p* < 0.001). Comparing patients with axSpA to those without, the pooled OR was 1.29 (95% CI 1.10–1.52; predictive interval 0.76–2.22; *p* = 0.001) for diabetes. The study suggests an increased prevalence and probably an increased risk of diabetes in people with axSpA. Routine screening for diabetes and lifestyle modifications should be encouraged in this cohort.

**Supplementary Information:**

The online version contains supplementary material available at 10.1007/s00296-024-05700-7.

## Introduction

Axial spondyloarthritis (axSpA) is a progressive inflammatory arthritis that affects the sacroiliac joints and typically emerges in the third decade of life. A wealth of evidence has demonstrated the rising number of comorbidities in people with inflammatory rheumatic conditions including axSpA [1, 2]. This has several negative consequences including higher mortality rates, increased healthcare expenditure and utilisation and reduction in quality of life of those affected [3–5]. AxSpA is a significant risk factor for cardiometabolic morbidity and mortality as demonstrated in a large study involving more than 22 million people. This risk is heightened in adults under 55 years with the potential to result in a disproportionate loss of years of life and disability [6]. Additionally, a large proportion of the observed risk is related to traditional cardiovascular risk factors. Previous studies have also shown that longer duration and more active disease further amplifies this risk [7].

Type 2 diabetes mellitus (T2DM) is a well-established risk factor for CVD. Several observational studies have investigated the relationship between inflammatory rheumatic diseases and diabetes; however, the results have been conflicting. Two recent systematic reviews have shown that both rheumatoid arthritis and systemic lupus erythematosus are associated with a significantly higher risk of diabetes compared to the general population [8, 9]. A recent UK population-based study has shown the risk of diabetes is two times higher in ankylosing spondylitis compared to the general population [6]. Similar results have been found in Taiwan [10]. Another systematic review which examined prevalence of comorbidities in axSpA patients did note a high prevalence of diabetes. However, diabetes was not the primary focus and only a small number of participants were assessed.

To date, there is no conclusive evidence on whether axSpA is associated with an increased risk of diabetes [10–12]. Furthermore, several relevant studies have been published since the previous analysis was conducted [13]. Improved understanding of this risk can help identify individuals at high risk and shape the development of targeted interventions for prevention and early detection. Therefore, the aim of this systematic review and meta-analysis was to: (i) determine the prevalence of diabetes in axSpA, and (ii) compare the risk of diabetes in axSpA populations and controls.

## Methods

This systematic review was conducted and reported in accordance with the Preferred Reporting Items for Systematic Review and Meta-Analysis (PRISMA) guidelines [14]. A protocol was registered in advance with PROSPERO (CRD42023482573). Ethical approval was not required.

### Search strategy

A search strategy was developed to identify observational studies that were published in MEDLINE, EMBASE and SCOPUS between 1st January 2000 and 15th November 2023. Briefly, the search strategy combined keywords related to the exposure (i.e. “axial spondyloarthritis” or “ankylosing spondylitis”) and outcome (i.e. “diabetes” or “type 2 diabetes”). A filter was applied to only include articles published in English. Details of the search strategy are reported in the Supplementary Data S1. Reference lists of retrieved articles were manually scanned to identify any relevant additional studies.

### Study selection

We used Rayyan (Qatar Computing Research Institute) for study screening. Following the PECOS (*population*,* exposure*,* comparator*,* outcome*,* study design*) framework, we included cross-sectional, prospective or retrospective longitudinal observational studies (*study design*) reporting on the prevalence, incidence or risk of type 1 and/or type 2 diabetes (*outcome*) in axSpA (*exposure*) in adult patients aged ≥ 18 years old (*population*). Any or no comparator was considered. We did not limit the inclusion of studies according to a specific definition of outcomes or exposures. Conference abstracts, editorials and systematic reviews were excluded.

Titles and abstracts were rigorously screened for inclusion by at least two reviewers (HS and PG). Subsequently, full text articles of the selected studies were evaluated. After reading the full text we included studies that met the inclusion criteria. Disagreements at all stages of the screening process were resolved by consensus of three reviewers (LG, HS and PG). We recorded the reason for the exclusion of all discarded studies.

### Data extraction

Data was then extracted by three reviewers (LG, HS, PG) into a standardised, pre-defined form. The following data was extracted from each study: title, author, publication year, country, study design, duration, population, comparator if any, total number of participants, patient demographics (age and sex) and details about how axSpA and diabetes were diagnosed. To assess our main outcome, we extracted additional data on the number of cases with and without diabetes among axSpA and non-axSpA groups.

### Risk of bias assessment

Quality assessment was conducted independently by two reviewers (HS and PG) for each study type using the JBI critical appraisal tool. Disagreement in study selection, data extraction or quality assessment was solved by consensus.

### Statistical analysis

Meta-analysis for each outcome was conducted in Stata 18.0. Then a narrative synthesis was done using effect size across all studies. A restricted maximum-likelihood random-effects model with the Knapp-Hartung/Sidik-Jonkman adjustment was used to estimate the pooled prevalence followed by the pooled odds ratio (OR) of diabetes in line with best practice guidelines [15]. We have reported summary statistics alongside the 95% confidence (CI) and predictive intervals in line with guidelines [16]. These results were further stratified by sex. Statistical inconsistency across studies was quantified using I^2^ and τ^2^ statistics. Small-study effects were assessed with funnel plots and Egger’s test.

## Results

### Characteristics of included studies

**Fig. 1:**
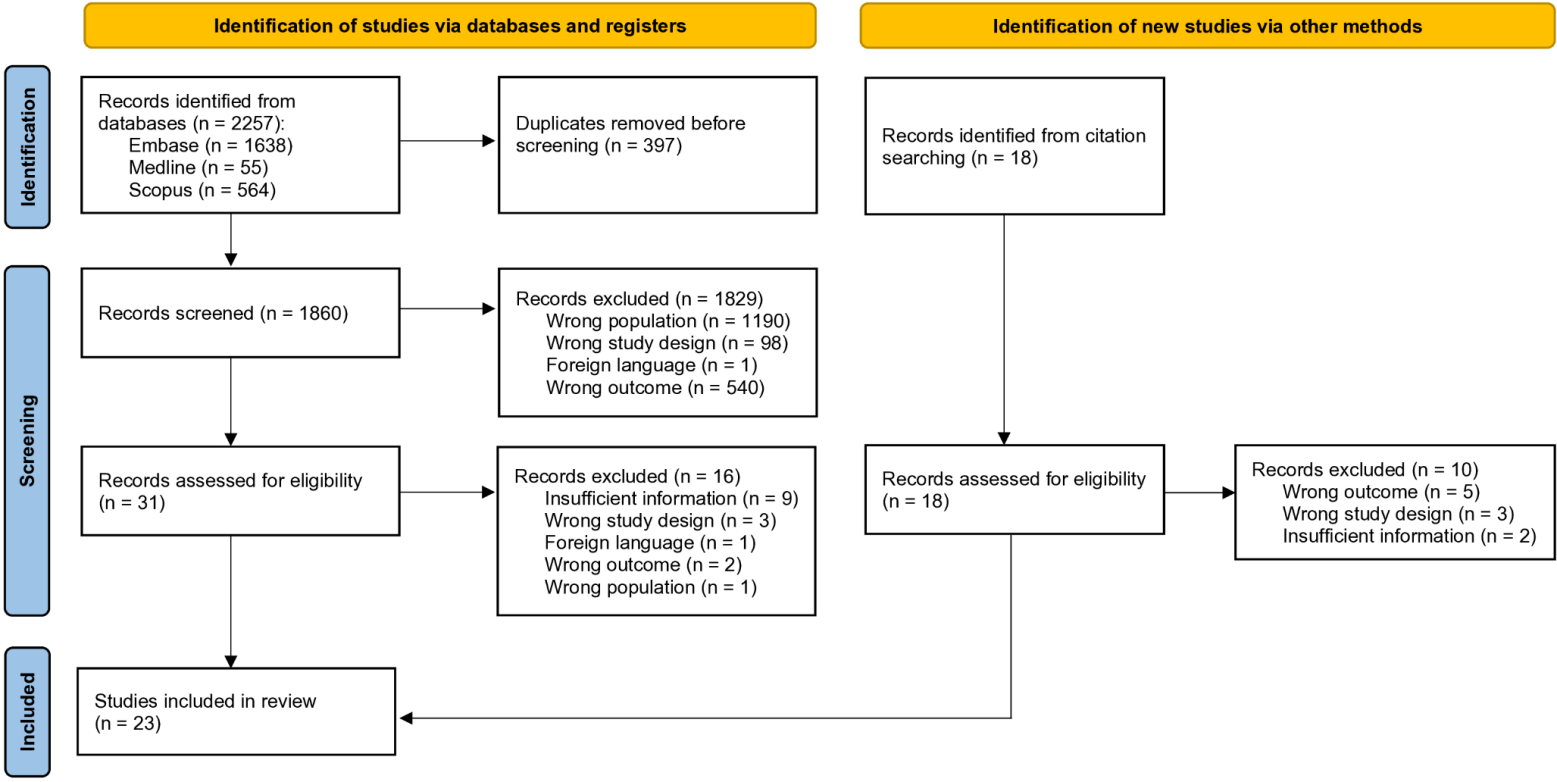
PRISMA flow diagram

A total of 2,257 studies were identified through the database search. After removal of duplicates, 1,860 studies underwent title and abstract screening during which 1,829 studies were excluded. A further 17 studies were identified from citation searching. We reviewed 49 observational studies for full-text screening, after which a further 26 were excluded and a total of 23 were included (Fig. 1). The most common reasons for exclusion were insufficient information or wrong outcome. Of the included studies, 10 were cross-sectional [11, 17–25], 8 were retrospective [5, 10, 26–31] and 5 were prospective [32–36].

The total number of patients included in the meta-analysis was 65 025. The main characteristics of included studies are shown in Table 1. The mean age of participants was 45.9 (range 31.2–59) years, and 63% (range 47.1 – 87.2%) were male. All studies were published after 2006 and most were population-based. 14 (61%) studies were conducted in UK/Europe. Sample sizes ranged from 94 to 21 473 participants. Studies were conducted in a diverse range of settings including large electronic health databases [26, 27, 31, 32], population registers [18, 19, 28, 33], insurance claims [10, 21, 29, 30, 35], national axSpA cohorts [24, 25] or regional hospitals/outpatient centres [5, 11, 17, 20, 22, 23, 34, 36]. AxSpA was defined and assessed differently across included studies (Table S3). Most studies used International Classification of Disease (ICD) codes, Assessment of Spondyloarthritis International Society (ASAS), modified New York criteria for identifying axSpA. The diagnosis of diabetes was mainly ascertained through medical records (Table S4). Lastly, the quality assessment scores were ≥ 7 across all 23 studies (Table S5).

**Table 1:** Characteristics of included studies

First Author (Year)	Country	Study design	Mean age (SD)	Males (%)	Follow-up (years)	Axial spondyloarthritis				
						Yes		No		
						DM Cases	DM Non-cases	DM Cases	DM Non-cases	
Ahmed (2016) [26]	UK	R	58.8(12.6)	87.2	10	10	84	33	343	
Bengtsson (2017) [33]	Sweden	P	50.0(13.9)	68.1	7	311	6137	10,118	256,317	
Bremander (2011) [34]	Sweden	P	52.3(14.8)	67.2	4	71	864	—	—	
Brophy (2012) [27]	UK	R	46.1(16.4)	75.9	11	187	1499	66,935	1,139,686	
Castaneda (2015) [35]	Spain	P	48.1(11.7)	72.9	10	56	682	34	643	
Cay (2023) [17]	Turkey	CS	—	70.6	—	63	1003	—	—	
Chen (2014) [13]	Taiwan	R	45.3(17.5)	53.0	5.8^†^	454	5364	—	—	
Cook (2018) [28]	UK	R	—	63.4	—	84	1170	203	4813	
Dregan (2017) [18]	UK	CS	37.0(13.0)	62.3	—	75	1325	—	—	
Gherghe (2015) [19]	France	CS	32.8(8.4)	47.1	—	6	639	—	—	
Han (2006) [29]	USA	R	47.3(12.0)	59.7	2	153	1690	523	6849	
Haroon (2015) [30]	Canada	R	45.6(15.9)	53.1	16	1820	19,653	5815	80,791	
Huang (2013) [36]	Taiwan	P	31.2(7.6)	73.8	3	75	4719	355	23,615	
Kang (2010) [31]	China	R	—	79.1	—	722	10,979	3444	55,061	
Landgren (2021) [20]	Sweden	CS	51.1(14.8)	56.4	—	28	559	—	—	
Mease (2019) [37]	USA	P	47.6(13.8)	63.7	3.1^†^	34	461	—	—	
Redeker (2020) [21]	Germany	CS	56.1	53.6	—	284	1492	—	—	
Stouten (2021) [32]	Belgium	R	41.0(15.0)	51.5	3	7	222	39	877	
Wibetoe (2017) [22]	Norway	CS	48.4(9.6)	64.9	—	28	807	—	—	
Yilmaz (2023) [23]	Turkey	CS	42.9(11.0)	52.9	—	74	489	72	491	
Zabotti (2021) [24]	Italy	CS	—	47.2	—	7	116	—	—	
Zhao (2019) [6]	UK	CS	45.5(14.3)	69.0	—	21	398	—	—	
Ziade (2020) [25]	Lebanon	CS	46.7(11.6)	56.3	—	14	89	—	—	

^†^Mean follow-up

—: Not available

R: retrospective; P: prospective; CS: cross-sectional; DM: diabetes mellitus

### Prevalence of diabetes

**Fig. 2:**
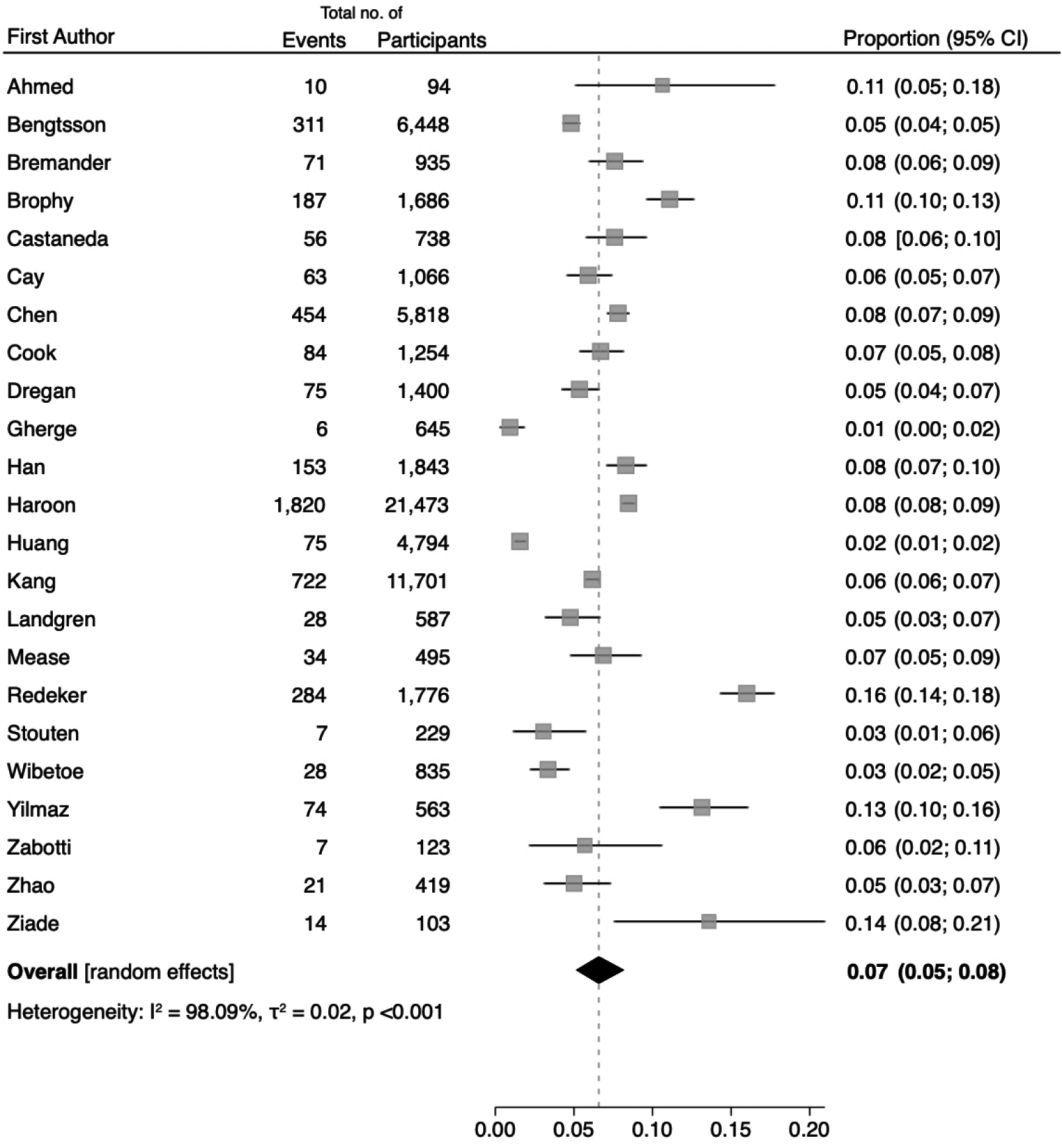
Pooled prevalence of diabetes in axSpA using random-effects model with the Knapp-Hartung adjustment

The prevalence of diabetes across the included studies ranged from 9 to 16%. The meta-analysis showed that the overall pooled prevalence of diabetes in axSpA was 7% (95% CI 5.9–8.0%) (Fig. 2). The overall predictive interval ranged from 2.4 to 12.9%. Four studies reported sex-specific prevalence of diabetes in axSpA. There was no clinical difference in the pooled prevalence of diabetes among males and females (Supplementary Data Figure S1). There was inconsistency across the included studies (I^2^ = 98.1% (95% CI 0.05–0.08), τ^2^ = 0.02). The funnel plot was symmetric suggesting no small-study effects (Figure S2).

### Risk of diabetes

**Fig. 3:**
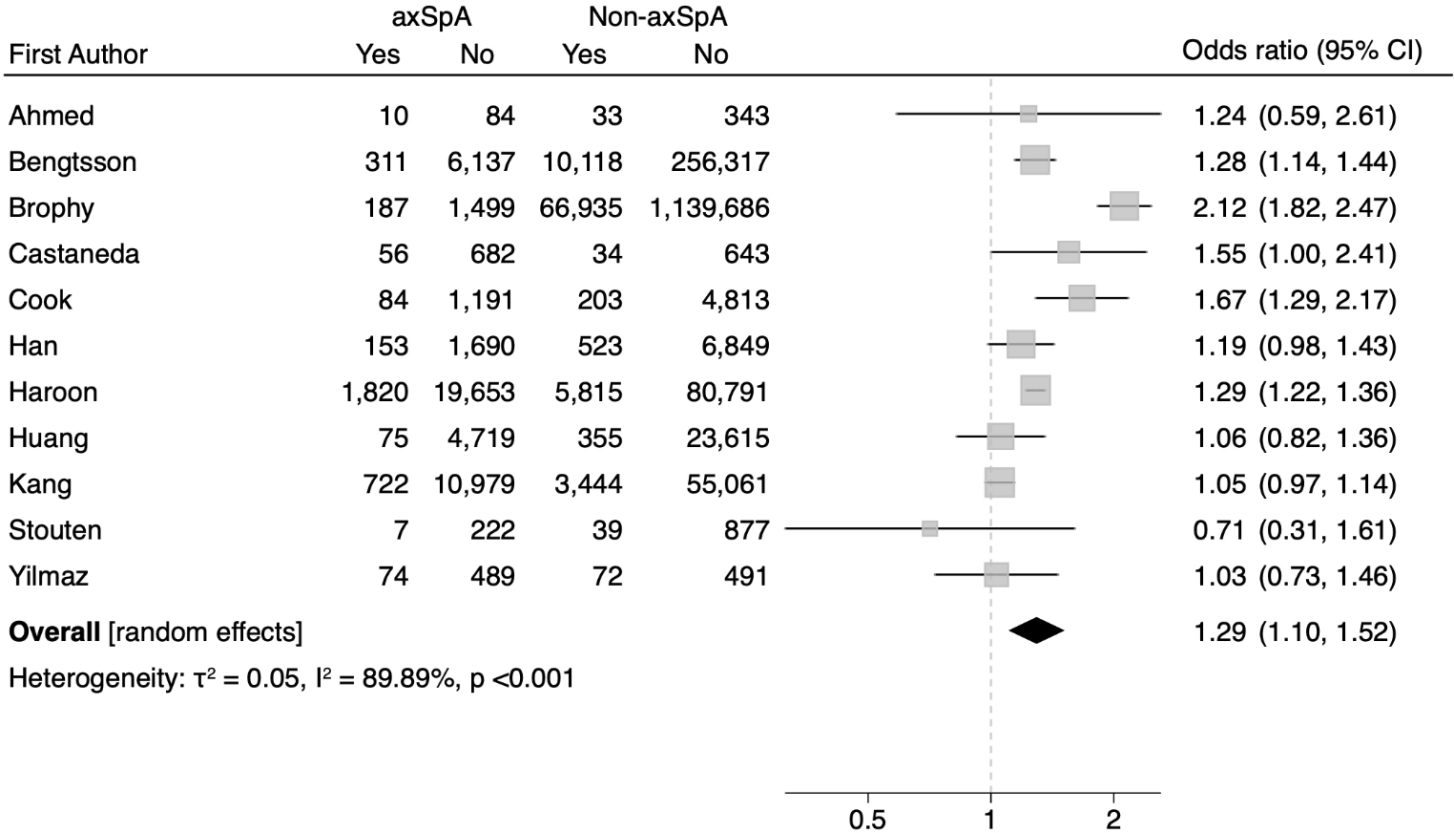
Pooled association between axSpA and diabetes using random-effects model with the Knapp-Hartung adjustment

A total of 11 studies including 50 823 participants reported on the association between axSpA and diabetes. Study specific OR ranged from 0.71 to 1.55 (Fig. 3). The pooled OR found a 1.29 (95% CI 1.10–1.52) greater risk of diabetes in participants with axSpA compared to participants without. The overall predictive interval was 0.76–2.22. Inconsistencies were noted across the studies (I^2^ = 89.9% (95% CI 0.10–0.42), τ^2^ = 0.05). However, there was no evidence of small-study effects (Figure S2).

## Discussion

The meta-analysis and narrative review including more than 65 000 participants reporting around 4500 cases of diabetes from 23 observational studies presents an overall picture on the high prevalence of diabetes in people with axSpA. We also found that people with axSpA compared to those without have an increased risk of developing diabetes.

Our findings are in line with other studies which have shown that comorbid conditions especially CVD and its risk factors such as diabetes, hypertension and obesity are highly prevalent among patients with axSpA compared to non-axSpA patients matched by age and sex [1, 7, 21, 37]. For example, the ASAS-COMOSPA study with 3984 people with axSpA across the globe found that 8.8% of participants also had diabetes [38] There are multiple reasons behind this observed risk, with a pivotal role played by inflammatory cytokines. Systemic inflammation and elevated pro-inflammatory cytokines are often observed in axSpA and have been shown to accelerate the process of atherosclerosis, obesity and related disorders such as metabolic syndrome, hypertension and dyslipidaemia [9, 39–41]. Recent evidence has also shown that dysregulation of NLRP3, an inflammatory signalling molecule can cause insulin resistance and subsequently increase the risk of diabetes and CVD [42]. Furthermore, axSpA patients particularly those with high disease activity are more likely to be physically inactive or immobile due to chronic pain, fatigue, spinal fusion or fractures [43]. Corticosteroids and non-steroidal anti-inflammatory drugs (NSAIDs) are commonly used in the management of AS. Studies have shown the heightened risk of hyperglycaemia and cardiovascular events with long-term use of these drugs [44, 45]. Disease modifying anti-rheumatic drugs (DMARDs) on the other hand may reduce the risk. Studies looking at diabetes in patients with rheumatoid arthritis have shown that DMARDs such as TNF inhibitors, hydroxychloroquine, and methotrexate may improve glycaemic control [41]. But less is known in the context of axSpA.

The results of our study add to the growing evidence that there is not only increased risk of CVD but also of common cardiovascular risk factors in patients with axSpA. Disentangling this risk further requires further research at various levels. Although our study showed the possibility of a causal relationship between axSpA and diabetes, there is a need for greater evidence from large prospective studies to confirm this. Our results also highlight the need for greater awareness and routine screening for diabetes in axSpA. We recommend that all clinicians involved in the care of axSpA patients should embrace every opportunity to educate them on their risk, provide lifestyle advice and check HbA1c. Furthermore, there is a growing need to understand the effects of conventional and biologic DMARDs on the risk of diabetes.

Our study has several strengths. Our search strategy was robust, and our data collection and quality assessment methods were rigorous. Each full-text article was reviewed by two authors. In addition, all the included studies were of good quality scoring at least 7 on the JBI critical appraisal tool. We performed a meta-analysis which provided a better estimate of the effect size and the generalisability of our findings. As observational associations could be biased by confounding factors, most estimates were adjusted in individual studies for several potential confounders. Limitations include that there was a high level of inconsistency among the included studies in terms of ascertainment of diabetes, the reference group and diagnosis of axSpA. These may have underestimated the prevalence of diabetes. However, our funnel plot was symmetric indicating a low risk of publication bias. Furthermore, most eligible studies did not provide a distinction between the different type of diabetes. Therefore, we cannot exclude the possibility that the data presented includes a sum of all types of diabetes. However, given that the mean age across the studies was 45 years, it is reasonable to conclude that the studies included in our meta-analysis focused on type 2 diabetes.

Despite the differences in the details of the included studies, taken as a whole, they appear to confirm that there is a high prevalence and possible increased risk of diabetes in patient with axSpA compared to non-axSpA controls. These results can be useful for patients and clinicians to better understand the relationship between axSpA and diabetes. They also demonstrate importance of routine screening for diabetes in people with axSpA. Furthermore, they can guide uptake of existing and development of new lifestyle interventions. Greater consistency across studies in the future and exploration of other factors like ethnicity will help further clarify the relationship between axSpA and diabetes.

## Electronic supplementary material

Below is the link to the electronic supplementary material.

Supplementary Material 1

## Data Availability

All data generated and analysed are included in the paper and supplementary data set.

## Declarations

### Disclaimer

As the corresponding author, I can confirm that no AI or editing software was employed for writing and editing this manuscript.
