# Supplementary material for: Diabetes in axial spondyloarthritis: a systematic review and meta-analysis of observational studies
Source: Rheumatol Int. 2024 Sep 12;44(11):2381–8. doi: 10.1007/s00296-024-05700-7 (PMC11424650; doi:10.1007/s00296-024-05700-7)

**APPENDIX**

**Diabetes in axial spondyloarthritis: a systematic review and meta-analysis**

Table of Contents

[Table S1: Search strategy 2](#_Toc167185105)

[Table S2: Data extraction template 4](#_Toc167185106)

[Table S3: Axial spondyloarthritis definitions 5](#_Toc167185107)

[Table S4: Diabetes mellitus assessment 6](#_Toc167185108)

[Table S5: JBI Critical Appraisal Scores 7](#_Toc167185109)

[Figure S1: Sex differences in prevalence 8](#_Toc167185110)

[Figure S2: Funnel plots 9](#_Toc167185111)

# **Table S1:** Search strategy

The following literature search terms were to identify English articles published between January 1, 2000 and November 15, 2023 in Medline, Embase and Scopus.

***Database: Medline***

| 1 | diabetes mellitus/ or diabetes mellitus type 1/ or diabetes mellitus type 2/ or "type 1 diabetes".mp. or "type 2 diabetes".mp. or "diabetes".mp. or "autoimmune diabetes".mp. or "brittle diabetes mellitus".mp. or "diabetes, autoimmune".mp. or "diabetes mellitus, brittle".mp. or "diabetes mellitus, insulin dependent".mp. or "diabetes mellitus, insulin-dependent".mp. or "diabetes mellitus, juvenile onset".mp. or "diabetes mellitus, ketosis prone".mp. or "diabetes mellitus, sudden onset".mp. or "diabetes mellitus, type 1".mp. or "diabetes mellitus, type i".mp. or "diabetes, type 1".mp. or "iddm".mp. or "insulin dependent diabetes mellitus 1".mp. or "insulin-dependent diabetes mellitus".mp. or "juvenile onset diabetes".mp. or "juvenile-onset diabetes mellitus".mp. or "ketosis-prone diabetes mellitus".mp. or "sudden-onset diabetes mellitus".mp. or "type 1 diabetes".mp. or "adult-onset diabetes mellitus".mp. or "diabetes, maturity-onset".mp. or "diabetes mellitus, adult onset".mp. or "diabetes mellitus, ketosis resistant".mp. or "diabetes mellitus, maturity onset".mp. or "diabetes mellitus, non insulin dependent".mp. or "diabetes mellitus, slow onset".mp. or "diabetes mellitus, stable".mp. or "diabetes mellitus, type 2".mp. or "diabetes mellitus, type ii".mp. or "diabetes, type 2".mp. or "ketosis-resistant diabetes mellitus".mp. or "mody".mp. or "maturity onset diabetes".mp. or "maturity onset diabetes mellitus".mp. or "niddm".mp. or "non-insulin-dependent diabetes mellitus".mp. or "slow-onset diabetes mellitus".mp. or "stable diabetes mellitus".mp. [mp=title, book title, abstract, original title, name of substance word, subject heading word, floating sub-heading word, keyword heading word, organism supplementary concept word, protocol supplementary concept word, rare disease supplementary concept word, unique identifier, synonyms, population supplementary concept word, anatomy supplementary concept word] | 794560 |
| --- | --- | --- |
| 2 | ankylosing spondylitis/ or axial spondyloarthritis/ or spondylitis/ or "axial spondyloarthritis".mp. or "spondylitis, ankylosing".mp. or "axspa".mp. or "spondyloarthropath*".mp. or "non-radiographic axial spondyloarthritis".mp. or "nr-axspa".mp. or "r-axspa".mp. or "sacroiliitis".mp. or "spondyloarthrit*".mp. or "spondylitis ankylos*".mp. [mp=title, abstract, heading word, drug trade name, original title, device manufacturer, drug manufacturer, device trade name, keyword heading word, floating subheading word, candidate term word] | 20609 |
| 3 | 1 and 2 | 236 |
| 4 | limit 3 to english language | 149 |
| 5 | limit 4 to yr="2000 -Current" | 55 |

***Database: Embase***

| 1 | diabetes mellitus/ or diabetes mellitus type 1/ or diabetes mellitus type 2/ or "type 1 diabetes".mp. or "type 2 diabetes".mp. or "diabetes".mp. or "autoimmune diabetes".mp. or "brittle diabetes mellitus".mp. or "diabetes, autoimmune".mp. or "diabetes mellitus, brittle".mp. or "diabetes mellitus, insulin dependent".mp. or "diabetes mellitus, insulin-dependent".mp. or "diabetes mellitus, juvenile onset".mp. or "diabetes mellitus, ketosis prone".mp. or "diabetes mellitus, sudden onset".mp. or "diabetes mellitus, type 1".mp. or "diabetes mellitus, type i".mp. or "diabetes, type 1".mp. or "iddm".mp. or "insulin dependent diabetes mellitus 1".mp. or "insulin-dependent diabetes mellitus".mp. or "juvenile onset diabetes".mp. or "juvenile-onset diabetes mellitus".mp. or "ketosis-prone diabetes mellitus".mp. or "sudden-onset diabetes mellitus".mp. or "type 1 diabetes".mp. or "adult-onset diabetes mellitus".mp. or "diabetes, maturity-onset".mp. or "diabetes mellitus, adult onset".mp. or "diabetes mellitus, ketosis resistant".mp. or "diabetes mellitus, maturity onset".mp. or "diabetes mellitus, non insulin dependent".mp. or "diabetes mellitus, slow onset".mp. or "diabetes mellitus, stable".mp. or "diabetes mellitus, type 2".mp. or "diabetes mellitus, type ii".mp. or "diabetes, type 2".mp. or "ketosis-resistant diabetes mellitus".mp. or "mody".mp. or "maturity onset diabetes".mp. or "maturity onset diabetes mellitus".mp. or "niddm".mp. or "non-insulin-dependent diabetes mellitus".mp. or "slow-onset diabetes mellitus".mp. or "stable diabetes mellitus".mp. [mp=title, book title, abstract, original title, name of substance word, subject heading word, floating sub-heading word, keyword heading word, organism supplementary concept word, protocol supplementary concept word, rare disease supplementary concept word, unique identifier, synonyms, population supplementary concept word, anatomy supplementary concept word] | 1410514 |
| --- | --- | --- |
| 2 | ankylosing spondylitis/ or axial spondyloarthritis/ or spondylitis/ or "axial spondyloarthritis".mp. or "spondylitis, ankylosing".mp. or "axspa".mp. or "spondyloarthropath*".mp. or "non-radiographic axial spondyloarthritis".mp. or "nr-axspa".mp. or "r-axspa".mp. or "sacroiliitis".mp. or "spondyloarthrit*".mp. or "spondylitis ankylos*".mp. [mp=title, abstract, heading word, drug trade name, original title, device manufacturer, drug manufacturer, device trade name, keyword heading word, floating subheading word, candidate term word] | 57501 |
| 3 | 1 and 2 | 1777 |
| 4 | limit 3 to english language | 1701 |
| 5 | limit 4 to yr="2000 -Current" | 1638 |

***Database: Scopus***

| 1 | “diabetes mellitus” OR “type 1 diabetes” OR “type 2 diabetes” OR “diabetes” OR “autoimmune diabetes” OR “brittle diabetes mellitus” OR “diabetes, autoimmune” OR “diabetes mellitus, brittle” OR “diabetes mellitus, insulin dependent” OR “diabetes mellitus, insulin-dependent” OR “diabetes mellitus, juvenile onset” OR “diabetes mellitus, ketosis prone” OR “diabetes mellitus, sudden onset” OR “diabetes mellitus, type 1” OR “diabetes mellitus, type i” OR “diabetes, type 1” OR “iddm” OR “insulin dependent diabetes mellitus 1” OR “insulin-dependent diabetes mellitus” OR “juvenile onset diabetes” OR “juvenile-onset diabetes mellitus” OR “ketosis-prone diabetes mellitus” OR “sudden-onset diabetes mellitus” OR “type 1 diabetes” OR “adult-onset diabetes mellitus” OR “diabetes, maturity-onset” OR “diabetes mellitus, adult onset” OR “diabetes mellitus, ketosis resistant” OR “diabetes mellitus, maturity onset” OR “diabetes mellitus, non insulin dependent” OR “diabetes mellitus, slow onset” OR “diabetes mellitus, stable” OR “diabetes mellitus, type 2” OR “diabetes mellitus, type ii” OR “diabetes, type 2” OR “ketosis-resistant diabetes mellitus” OR “mody” OR “maturity onset diabetes” OR “maturity onset diabetes mellitus” OR “niddm” OR “non-insulin-dependent diabetes mellitus” OR “slow-onset diabetes mellitus” OR “stable diabetes mellitus” | 1194082 |
| --- | --- | --- |
| 2 | “axial spondyloarthritis” OR “spondylitis, ankylosing” OR “axspa” OR “spondyloarthropath*” OR “non-radiographic axial spondyloarthritis” OR “nr-axspa” OR “r-axspa” OR “sacroiliitis” OR “spondyloarthrit*” OR “spondylitis ankylos*” | 30142 |
| 3 | 1 and 2 | 689 |
| 4 | limit 3 to english language | 638 |
| 5 | limit 4 to year="2000 -Current" | 564 |

# **Table S2**: Data extraction template

Data was extracted on the elements listed in Table S2 for all identified studies.

| - First author - Publication year - Study design - Country - Study duration - Study population - Comparator | - Sample size - Primary outcome - Variables adjusted for - Definition and assessment of axSpA - Definition and assessment of diabetes - Participant demographics (age, gender) - Number of cases of diabetes |
| --- | --- |

# **Table S3:** Axial spondyloarthritis definitions

| First Author (Year) | Axial spondyloarthritis definition and assessment | Comparator |
| --- | --- | --- |
| Ahmed (2016) | Aged ≥18 years with AS between 1999 and 2009 | Non-inflammatory diseases matched by year of birth, gender and general practice from the same database |
| Bengtsson (2017) | At least one physician visit with AS diagnosis in outpatients between 2001 and 2009. Diagnosis based on ICD-10 code M45 and ICD-9 code 720 | Controls from the same database matched based on sex, age and geography |
| Bremander (2011) | Clinic visit with diagnosis of AS between January 2004 and December 2007 | General population seeking healthcare |
| Brophy (2012) | AS identified using read codes | Non-AS from the same database |
| Castaneda (2015) | AS patients attending rheumatology clinics between July 2010 and January 2012. Diagnosis based on modified New York criteria | Age and sex matched controls without chronic inflammatory rheumatic diseases |
| Cay (2023) | axSpA diagnosis between February 2019 and December 2020. Diagnosis based on ASAS or modified New York criteria | — |
| Chen (2014) | People with at least three medical visits for AS or SpA between 2000 and 2010. Diagnosis based on ICD-9 code 720.0 | Non-AS from the same database |
| Cook (2018) | AS identified using read codes | Non-inflammatory diseases from same database |
| Dregan (2017) | AS identified using read codes | Non-inflammatory diseases from same database |
| Gherghe (2015) | Aged 18-50 years with inflammatory back pain for a period of 3 months to 3 years. Recruited between October 2007 and April 2010. Diagnosis based on ASAS, Amor criteria, modified New York criteria or European Spondyloarthropathy Study Group criteria | General population aged 18-74 years from a national survey in 2006 |
| Han (2006) | Age >17 years with AS between January 2001 and December 2002. Diagnosis based on ICD-9 code 720.0 | Controls from the same database matched based on sex, age, geography and cardiovascular disease risk factors |
| Haroon (2015) | AS for more than 2 years between April 1992 and December 2011. Diagnosis based on ICD-10 code M45 and ICD-9 code 720 | Controls without AS from same database matched based on sex and age |
| Huang (2013) | Age 18-45 years and two visit diagnoses with AS between January and December 2001; identified using read codes | Controls without AS from same database matched based on sex and age |
| Kang (2010) | AS based on ICD-9 code 720 or 720.0 | Controls from same database matched on sex, age, income and urbanisation |
| Landgren (2021) | Aged ≥18 years with AS between January 2015 and February 2017. Diagnosis based on CD-10 code M459 | Age matched controls |
| Mease (2019) | Aged ≥18 years with axSpA between March 2013 and March 2018. Diagnosis using read codes | — |
| Redeker (2020) | axSpA in at least two quarters of the year 2014; diagnosis using ICD-10 code M45 | Age and sex matched controls without axial SpA from the same database |
| Stouten (2021) | AS diagnosed between 1999 and 2012. Diagnosis using ICPC code L99 and verified by rheumatologists as having a diagnosis of SpA | Non-musculoskeletal disease matched based on gender, type of GP practice and year of diagnosis from the same database |
| Wibetoe (2017) | Age 30-80 years with axSpA from rheumatology clinics | Other inflammatory joint diseases |
| Yilmaz (2023) | AS diagnosed between December 2018 and March 2021 in a government hospital. Diagnosis based on ASAS or modified New York criteria | Chronic low back pain due to intervertebral disc prolapse matched based on age and gender |
| Zabotti (2021) | Adults >18 years with new diagnosis of SpA between June 2017 and June 2019 and naïve to DMARDs. Diagnosis based on ASAS criteria | — |
| Zhao (2019) | axSpA with comorbidities attending tertiary centre between 2010 and 2017. Diagnosis based on ASAS criteria | Isolated axSpA (without comorbidities) |
| Ziade (2020) | Adults >18 years with axSpA attending rheumatology clinics between 2016 and 2018 | — |

Studies are sorted by first author name.

AS: ankylosing spondylitis; axSpA: axial spondyloarthritis; SpA: spondyloarthritis.

# **Table S4:** Diabetes mellitus assessment

| First Author (Year) | Diabetes mellitus assessment |
| --- | --- |
| Ahmed (2016) | Primary care database |
| Bengtsson (2017) | ICD codes within primary care database |
| Bremander (2011) | Population register |
| Brophy (2012) | Primary care database |
| Castaneda (2015) | — |
| Cay (2023) | Self-reported |
| Chen (2014) | Health insurance database |
| Cook (2018) | Population register |
| Dregan (2017) | Population register |
| Gherghe (2015) | DESIR cohort and population register |
| Han (2006) | Health insurance database |
| Haroon (2015) | Regional health database |
| Huang (2013) | Health insurance database |
| Kang (2010) | Health insurance database |
| Landgren (2021) | Self-reported |
| Mease (2019) | Regional database |
| Redeker (2020) | Health insurance database |
| Stouten (2021) | Primary care database |
| Wibetoe (2017) | Self-reported |
| Yilmaz (2023) | Medical records |
| Zabotti (2021) | Medical records |
| Zhao (2019) | Medical records |
| Ziade (2020) | Medical records |

Studies are sorted by first author name.

# **Table S5:** JBI Critical Appraisal Scores

| First Author (Year) | Study design | Score based on appropriate JBI appraisal* | | | | | | | | | | | | |
| --- | --- | --- | --- | --- | --- | --- | --- | --- | --- | --- | --- | --- | --- | --- |
|  |  | **1** | **2** | **3** | **4** | **5** | **6** | **7** | **8** | **9** | **10** | **11** | **Total** |  |
| Ahmed (2016) | Retrospective study | Y | Y | Y | Y | Y | NA | Y | Y | Y | NA | Y | 9/11 |  |
| Bengtsson (2017) | Prospective study | Y | Y | Y | Y | N | Y | Y | Y | Y | NA | Y | 9/11 |  |
| Bremander (2011) | Prospective study | Y | Y | Y | Y | Y | Y | Y | Y | N | N | Y | 9/11 |  |
| Brophy (2012) | Retrospective study | Y | Y | Y | Y | Y | Y | Y | Y | Y | Y | Y | 11/11 |  |
| Castaneda (2015) | Prospective study | Y | Y | Y | Y | Y | Y | Y | Y | Y | Y | Y | 11/11 |  |
| Cay (2023) | Cross-sectional | Y | Y | Y | Y | Y | Y | Y | Y | NA | NA | NA | 8/8 |  |
| Chen (2014) | Retrospective study | Y | Y | Y | Y | N | Y | Y | Y | Y | N | Y | 9/11 |  |
| Cook (2018) | Retrospective study | Y | Y | Y | Y | Y | Y | Y | Y | N | N | Y | 9/11 |  |
| Dregan (2017) | Cross-sectional | Y | Y | Y | Y | Y | Y | Y | Y | NA | NA | NA | 8/8 |  |
| Gherghe (2015) | Cross-sectional | Y | Y | Y | Y | Y | Y | Y | Y | NA | NA | NA | 8/8 |  |
| Han (2006) | Retrospective study | Y | Y | Y | Y | Y | Y | Y | Y | N | N | N | 8/11 |  |
| Haroon (2015) | Retrospective study | Y | Y | Y | Y | Y | Y | Y | Y | Y | Y | Y | 11/11 |  |
| Huang (2013) | Prospective study | Y | Y | Y | Y | Y | Y | Y | Y | Y | Y | Y | 11/11 |  |
| Kang (2010) | Retrospective study | Y | Y | Y | Y | N | Y | Y | N | U | U | Y | 7/11 |  |
| Landgren (2021) | Cross-sectional | Y | Y | Y | Y | Y | Y | Y | Y | NA | NA | NA | 8/8 |  |
| Mease (2019) | Prospective study | Y | Y | Y | N | N | Y | Y | Y | U | U | Y | 7/11 |  |
| Redeker (2020) | Cross-sectional | Y | Y | Y | Y | Y | Y | Y | Y | NA | NA | NA | 8/8 |  |
| Stouten (2021) | Retrospective study | Y | Y | Y | Y | Y | Y | Y | Y | N | N | Y | 9/11 |  |
| Wibetoe (2017) | Cross-sectional | Y | Y | Y | Y | Y | Y | Y | Y | NA | NA | NA | 8/8 |  |
| Yilmaz (2023) | Cross-sectional | Y | Y | Y | Y | Y | Y | Y | Y | NA | NA | NA | 8/8 |  |
| Zabotti (2021) | Cross-sectional | Y | Y | Y | Y | Y | Y | Y | Y | NA | NA | NA | 8/8 |  |
| Zhao (2019) | Cross-sectional | Y | Y | Y | Y | Y | Y | Y | Y | NA | NA | NA | 8/8 |  |
| Ziade (2020) | Cross-sectional | Y | Y | Y | Y | Y | Y | Y | Y | NA | NA | NA | 8/8 |  |

*Y = Yes; N = No; U = Unclear; NA = not applicable.

# **Figure S1:** Sex differences in prevalence

1. ***Females***


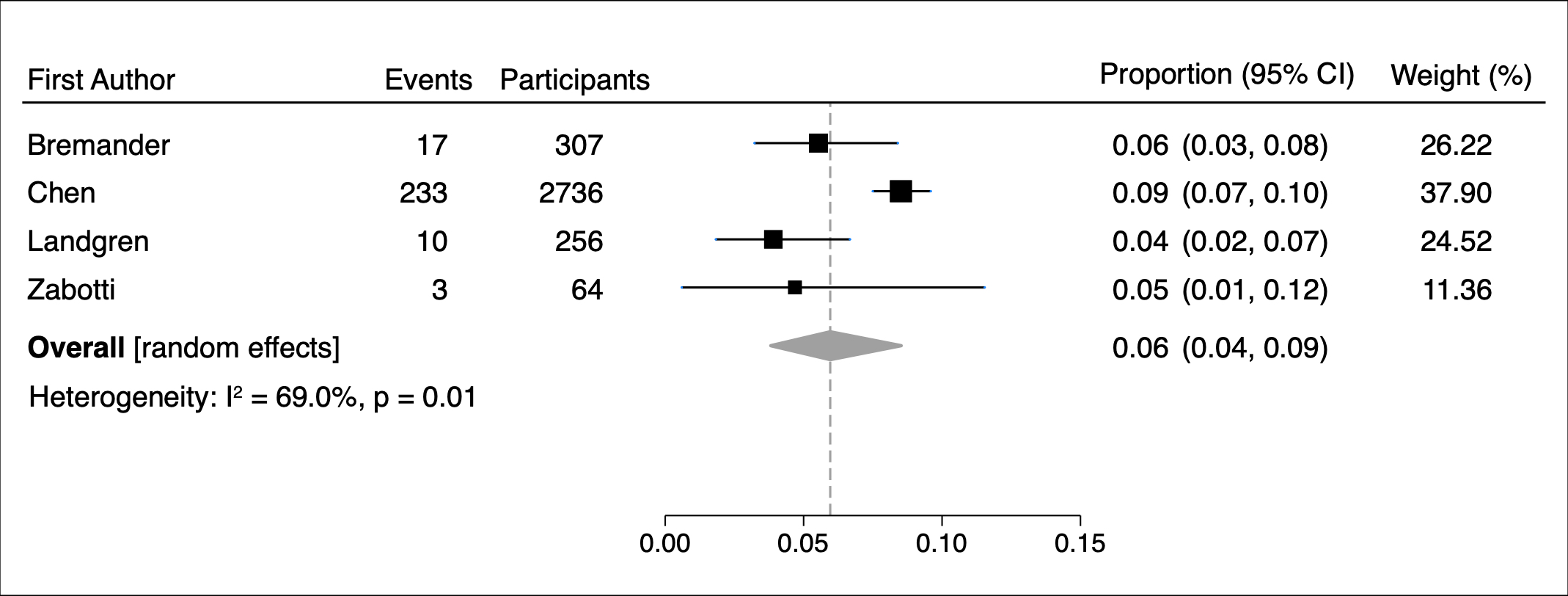


1. ***Males***


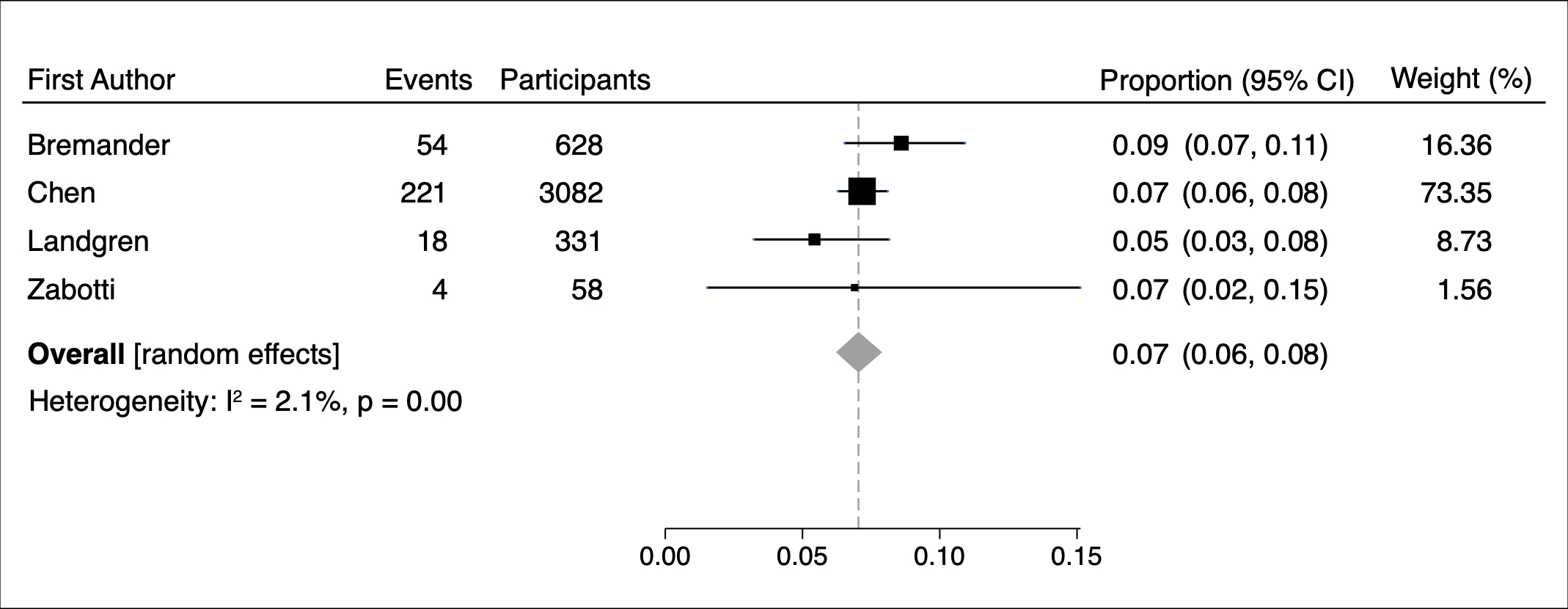


# **Figure S2:** Funnel plots


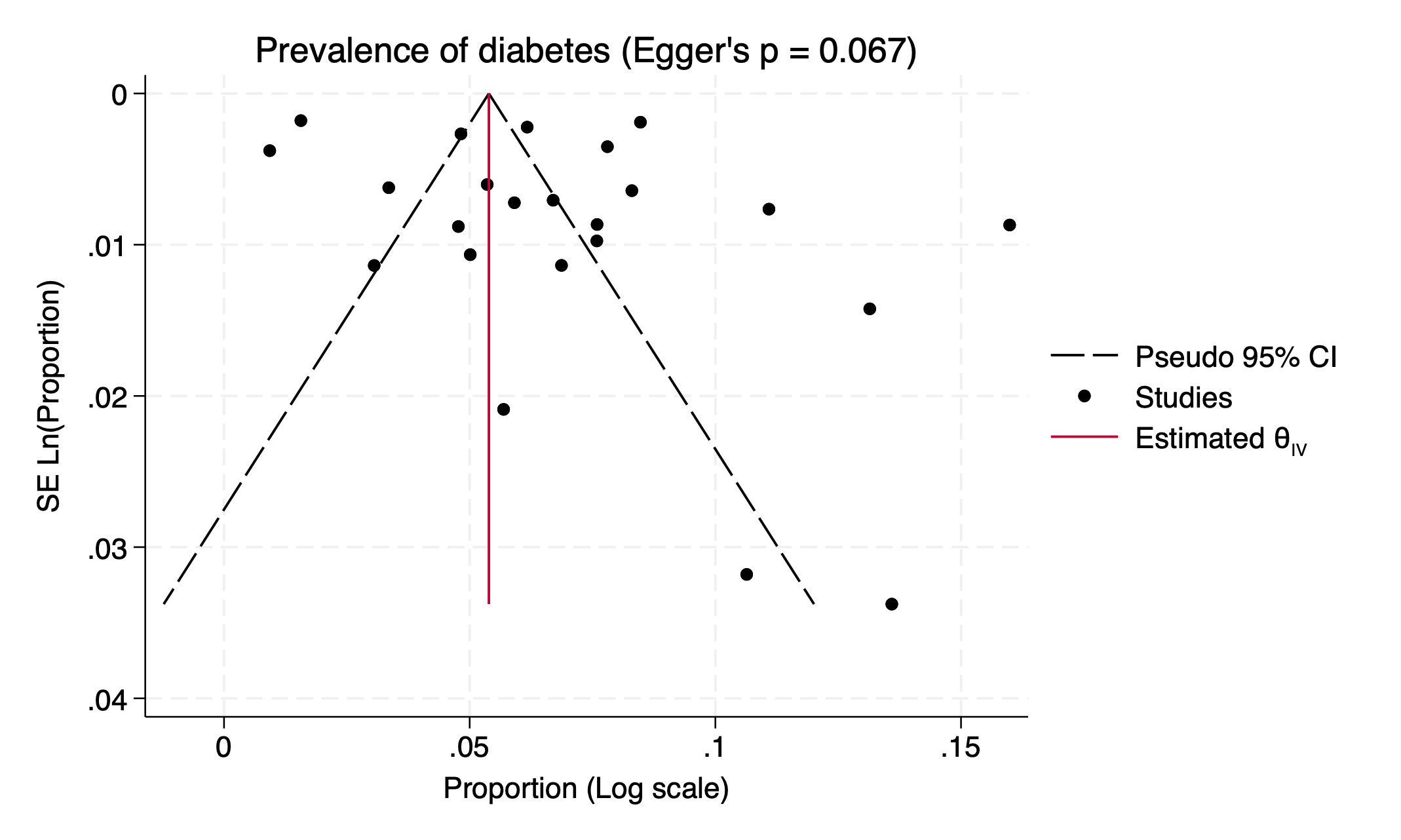

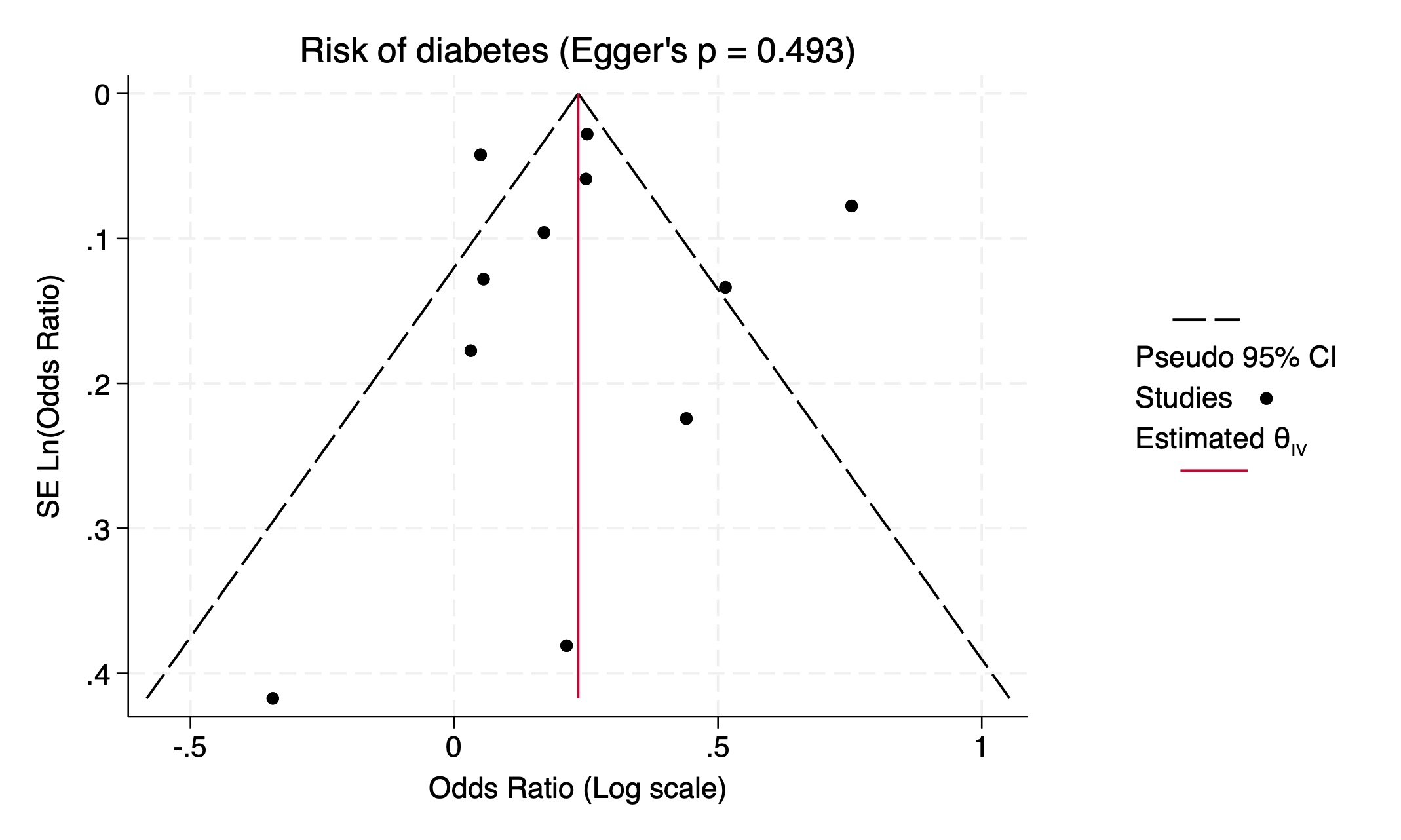

Supplement: Supplementary file 1 — Supplementary Material 1 [file 296_2024_5700_MOESM1_ESM.docx]
